# Effect of exfoliation corrosion on the efficient hybrid joint of AA2024-T3 and AA2198-T8 formed by friction stir welding

**DOI:** 10.1016/j.heliyon.2023.e16577

**Published:** 2023-05-29

**Authors:** Ahmed Samir A. Alemdar, Shawnim R. Jalal, Mohammedtaher M. Mulapeer

**Affiliations:** Mechanical & Mechatronics Dept., College of Engineering, Salahaddin University-Erbil (SUE), Iraq

**Keywords:** Aluminum, Alloy, Pitting corrosion, Exfoliation corrosion, Welding

## Abstract

The third-generation alloy, AA2198-T8, is highly recommended for the aerospace industry. However, it has come under scrutiny due to its high cost. This study aims to decrease the cost of manufacturing through a hybrid design that uses AA2198-T8 alloys for the crucial parts and AA2024-T3 alloys for the remaining structure. The two main techniques for joining AA2024-T3 to AA2198-T8 are reversed double-sided friction stir welding (DS-FSW) and traditional single-sided friction welding (SS-FSW). They were carried out under unaltered tool rotation speed followed by five different welding speeds. The mechanical properties of the joints were explored, and the maximum joining efficiency of the welding process was 96% for reversed DS-FSW at 102 mm/min welding speed. The hybrid joint was tested for compliance with ASTM G34 standards to examine the welding joint's exfoliation corrosion (EXCO) for eight exposure times. The findings showed that joint efficiency decreased relative to as-welded joints, and mechanical property deterioration increased with EXCO exposure duration, reaching 40% for specimens exposed for 120 h to the corroding solution. It has been noted that morphology and grain size changes significantly impact EXCO.

## 1 Introduction

In the past few decades, the application of aluminum alloys in the industrial sector has increased by 90% [[1], [2], [3], [4], [5], [6]]. Despite the thickness reduction, flashes and keyholes are formed in FSW joints [7]. The process of joining dissimilar materials via friction stir welding (FSW) has become one of the potent solutions for rapid development in the industrial sector, especially for aluminum and its alloys. FSW has been a successful green joining process, especially for aluminum alloys. Aluminum alloys have several advantages over traditional iron-based alloys, including low density, resistance to corrosion, and superior thermal and electrical conductivity. These alloys are in high demand, particularly in the aerospace industry, because some are produced in the soft state and heat-treated to a hardness equivalent to that of structural steel [[8], [9], [10], [11]]. The new third-generation Al–Cu–Li alloys, such as the AA2198-T8 alloy, are being investigated as a potential replacement for the AA2024 alloy in aerospace fuselage because of its good mechanical properties [10]. The third generation Al–Li alloys were developed for various uses, such as military, space, and commercial fuselages. These alloys have better mechanical properties than the typical 2xxx and 7xxx series alloys, such as a higher strength-to-weight ratio [12,13]. They provide the transportation sector with certain primary benefits that make them desirable [14]. Lithium is among the lightest metallic elements, with a 534 kg/m^3^ density. Lithium has a 2700 kg/m3 density and can be alloyed with aluminum to lower its density. In reality, the density of Al is lowered by 3%, or nearly 80 kg/m^3^, for every 1% weight of Li added. In addition, lithium is a unique metal that, when added to aluminum, enhances its elastic modulus by about 6%, or nearly 2.5 GPa per 1% weight of Li added [15]. As a result, their specific strength and modulus are relatively high. These alloys can be produced in the current production equipment since their behavior during extrusion, forging, machining, and shaping is similar to standard aluminum alloys. Despite these advantages, it has been found that these alloys are susceptible to specific types of localized corrosion because of the high reactivity of Li and the subsequent intermetallic phases that include Li [16].

This study aims to decrease production costs for industrial applications and set the most efficient welding parameters for hybrid joints. The aerospace industry highly recommends using third-generation Al–Li–Cu alloys, namely the AA2198-T8 alloy. It is a reasonable choice to use AA2198-T8 alloys for the critical areas of the construction and AA2024-T3 alloys for the remaining portions of it. As a result, green and practical joints have been made using reversed double-sided and single-sided FSW. The four unique zones that emerge from severe plastic deformation during FSW are the stir zone (SZ), thermomechanically affected zone (TMAZ), heat affected zone (HAZ), and base metal (BM). The impact of the exfoliation corrosion is different in these zones, especially at the dissimilar joints. Therefore, the dissimilar joint has a heterogeneous microstructure due to the BM's placement on the advancing side (AS) and retreating side (RS). On the other hand, exfoliation corrosion usually affects welded alloys and rolled plates in high-strength aluminum alloys used in aircraft fuselages [[16], [17], [18]]. Exfoliation is caused by exposed end grains on the countersink and hole bore edges, frequently observed on the upper wing skins around the holes [20].

## 2 Materials and methods

**Fig. 1:**
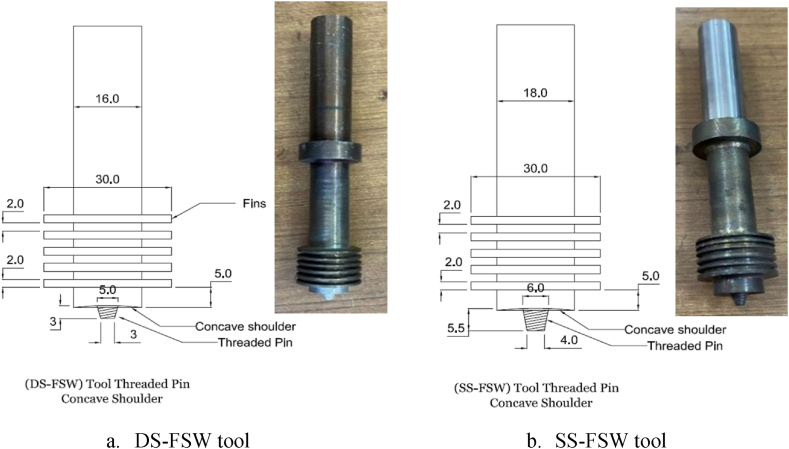
Dimensions of the DS-FSW and SS-FSW tools, all dimensions in (mm).

In this work, two base metals from traditional aluminum-copper alloys, AA2024-T3, and third-generation aluminum-copper lithium alloys, AA2198-T8 (300 mm × 75 mm × 6 mm), were combined employing the two FSW process methods, reversed DS-FSW and SS-FSW. Spectro Maxx machine was used to analyze the chemical composition of the base materials AA2198-T8 and AA2024-T3, and the results are shown in Table 1. This study used the (NK-IWASHITA) model type of the milling machine to execute FSW. The joining process parameters for both welding techniques are illustrated in Table 2. Dissimilar plates were clamped to the stiff base plate (backing plate) to prevent sliding and shifting while the FSW operations were performed. The FSW tool was made of hot-worked AISI H13 steel, which was examined and found suitable for friction stir welding with aluminum alloys. Though, after heat treatment, the measured hardness value of the tool was found to be 66 HRC [21]. The geometry of the tools for both techniques was tapered threaded pins with concave shoulders [22,23]. Two types of FSW hybrid materials joints were formed using dissimilar pin and shoulder sizes for different material penetration and friction stir welding processes. Fig. 1 (a,b) illustrates the reversal of passing DS-FSW and SS-FSW, respectively. Thus, the design of both techniques is integrated with the fin sections for enhancing tool life and avoiding the tool from heating up. The goal of the study is to choose between the traditional SS-FSW and the reversed (advanced side AS and retreating side RS) DS-FSW as the most appropriate FSW method. For this, the five distinct running welding speeds of 36, 76, 102, 146, and 216 mm/min were investigated. The welding parameters, namely the tool rotation speed, tilt angle, plunge-in depth, and dwell time (DT), were kept constant at 580 rpm, 2°, 0.3 mm, and 20 s, respectively. To perform a clean welding operation out of oxides and dirts, 0.3 mm removed from the base material's butt faces and swabbed them with ethanol before the joining process via reversed DS-FSW and SS-FSW [24].

**Table 1:** Chemical composition of BM AA2198-T8 and AA2024-T3 (wt.%).

Element	Cu	Li	Fe	Mg	Mn	Si	Ti	Ag	Zn
AA2198-T8	3.4	1.02	0.08	0.75	0.3	0.05	0.07	0.5	0.3
AA2024-T3	3.83	–	0.45	1.84	0.75	0.5	0.12	0.5	0.23

**Table 2:** Welding process parameters for fabricated hybrid joints.

Welding Process Parameters	SS-FSW	Reversed DS-FSW
Tool shoulder diameter (mm)	18	16
Pin length (mm)	5.5	3
Pin diameter (mm)	6–4	5–3
Tool tilt angle, degree	2°	2°
Pin shape	Tapered Threaded Cylindrical TTC	Tapered Threaded Cylindrical TTC
Welding speed (mm/min)	36, 76, 102, 146, and 216	36, 76, 102, 146, and 216
Dwell time (sec)	20	20

**Fig. 2:**
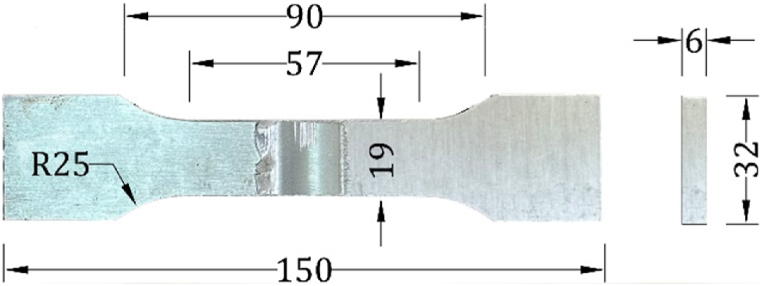
Dimensions of the tensile sample, all dimensions are in “mm.”

In this investigation, the joints obtained through reversed DS-FSW and SS-FSW were passed through several crucial tests according to particular international standards. Three fabricated hybrid joints were prepared for each selected parameter and welding condition. As an essential part of the work, mechanical tests were conducted to identify the joint weld parameter. According to the American Welding Society standard AWS D17.3/17.3 M 2016 specification, the tensile specimens were sectioned and machined to the required dimensions, as shown in Fig. 2. The joint efficiency of the tensile strength test is considered by comparing the ultimate tensile strength UTS of the AA2024-T3 base material. A Vickers Hardness digital automatic tester machine was used to measure the microhardness tests HV05 profile according to ASTM E92 guideline standards. The macro-section of the fabricated hybrid joints revealed via the polishing sections were etched using Flick's macro etchant consisting of 15 ml HCl, 10 ml HF, and 90 ml H_2_O, washed in warm water, followed by dipping in HNO_3_. The fractures with the ultimate strength were considered, and the most efficient joint was selected for further tests. While revealing the typical microstructure conforming to the ASTM E3 standard, the double etchant was employed after grinding and polishing using grit papers and diamond pastes to remove minor scratches on the surface. Thus, for etching purposes, two solutions were arranged; Solution A consisted of 25 ml of HNO_3_ and 75 ml of Distilled Water (H_2_O) (DW); Solution B consisted of 0.5 g of NaF, 1 ml HNO_3_, 2 ml HCL, and 97 ml H_2_O. The prepared specimen surface was dipped for 60 s in solution A at 70 °C and cooled sufficiently using cold water, then engrossed in solution B for 30 s and splashed in a stream of warm water. The exfoliation corrosion test was used for testing the joint benchmark efficiency for eight different aging times as per ASTM G34. The prepared reagent solution consisted of 234 g of NaCl and 50 g of KNO_3_ mixed in 1 L of W), which added 6.3 ml of concentrated HNO_3_. Thus, the solution had a pH of 0.4 and was maintained at a room temperature of 25 ± 3 °C. The macro capture of the attack EXCO surfaces was classified as ASTM G34, and the tensile test for different exposure times was conducted according to AWS D17.3/17.3 M standard, 2016. Furthermore, the X-Ray Diffraction (XRD) examination was conducted to measure the degree of crystallinity of the benchmark joint of the as-welded specimen. The fracture surfaces of the joint were tested using a scanning electron microscope (SEM) to reveal and study the formation of defects and failure points.

## 3 Results & discussions

### 3.1 Mechanical properties

**Fig. 3:**
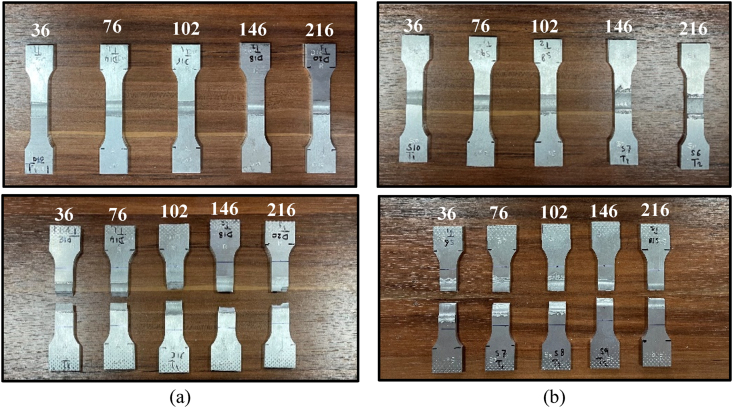
Tensile Samples before and after testing for various welding speeds of (a) reversed DS-FSW and (b) SS-FSW.

The efficiency of the hybrid joint was assessed using the parameters for both welding methods (reversed DS-FSW and SS-FSW) for ultimate tensile strength (UTS), yield strength (YTS), and percentage elongation (EL). The specimens for the tensile test were sliced and machined perpendicular to the welding line and centered in the specimen gauge, as shown in Fig. 3. A 50 mm ± 10 mm external Instron extensometer was mounted on the specimen's cross-section shortened gauge length. The outcomes of the tensile tests showed that the SS-FSW tensile strengths were significantly lower than that of the base material AA2024-T3. The welding speed had a crucial effect on the tensile strength and the microhardness in distinct zones of the weldment. While raising the welding running speeds from 36 mm/min to 216 mm/min of unaltered tool rotation speed at 580 rpm in two welding processes, the tensile strength increased and decreased for both welding processes (reversed DS-FSW and SS-FSW).

**Fig. 4:**
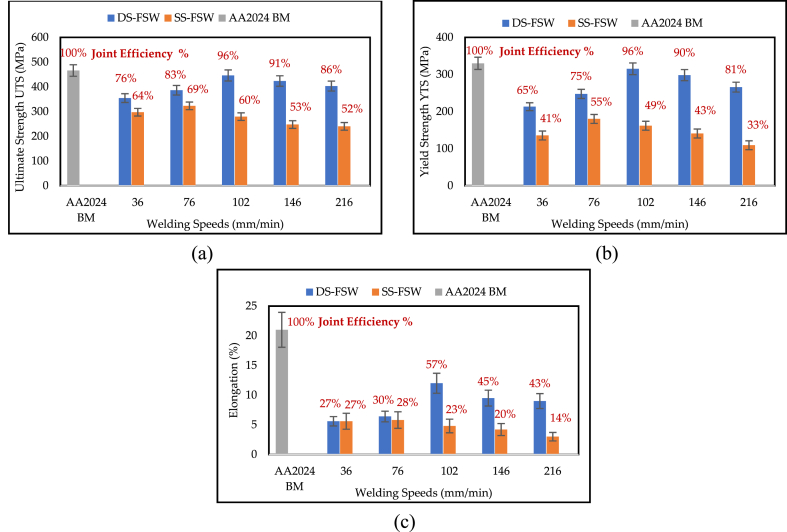
Tensile properties of reversed DS-FSW and SS-FSW (a) UTS, (b) YTS, and (c) EL.

The fracture locations of all joints were obtained by tensile tests conducted at five different welding speeds. Fig. 3 (a) shows that the joints produced in the reversed DS-FSW at 36 mm/min break in the stir zone and have the lowest tensile strength. However, the highest tensile strength was accomplished at 102 mm/min welding running speed. Meanwhile, the fracture was in the TMAZ with evident necking phenomena. The fracture in tensile specimens of the SS-FSW fractured in the SZ, as shown in Fig. 3 (b). Except for the 76 mm/min welding speed, the fracture locations were in the TMAZ. Thus, the maximum joining efficiency of 96% in the mechanical characteristics was recorded in DS-FSW at a welding running speed of 102 mm/min. The comparison with the base metal of AA2024-T3 is shown in Fig. 4(a–c). The extreme UTS, YTS, and EL were 446 MPa, 315.2 MPa, and 12%, correspondingly. The corresponding minimum values obtained at 36 mm/min were 354.5 MPa, 213.1 MPa, and 5.6%. For the SS-FSW hybrid joints, the extreme UTS, YTS, and EL found at 76 mm/min were 323 MPa, 180.1 MPa, and 5.8%, respectively. The efficiency of SS-FSW welding did not exceed 69%.

**Fig. 5:**
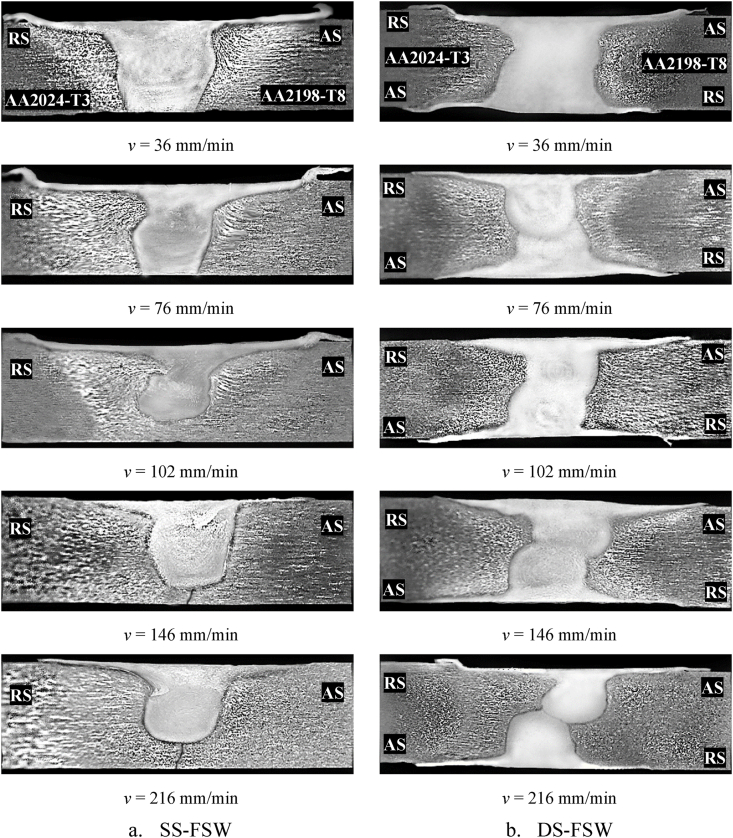
Macro-section of the SS-FSW and reversed DS-FSW for five different welding speeds.

Fig. 5 (a,b) presented the macro-sections of the effect of the different travel speeds with unaltered spindle speeds of 580 rpm for SS-FSW and reversed DS-FSW. Meanwhile, in the SS-FSW process at 146 and 216 mm/min welding speeds, the macro-section revealed that the bottom defects begin to be observed, which indicates insufficient compatibility of the welding speeds with the tool rotation speeds to produce sufficient joining heat. On the other hand, in reversed DS-FSW misalignment defect was observed at the same travel speeds. Marco-section examination indicates weak fractured locations in the tensile test and reveals the sound weld between hybrid joints for both welding processes.

**Fig. 6:**
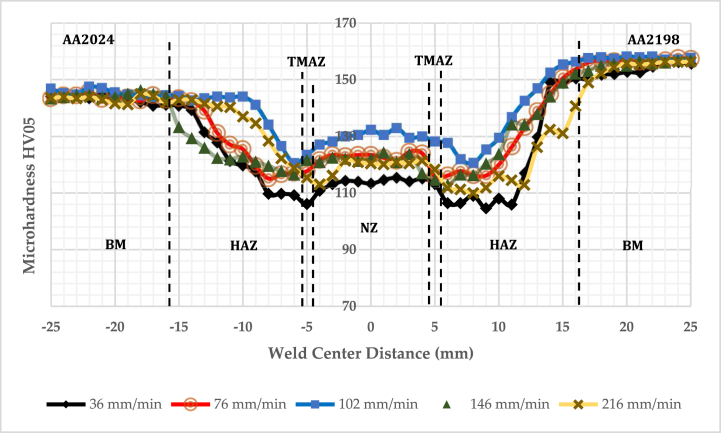
Microhardness of reversed double-sided friction stir welding DS-FSW.

**Fig. 7:**
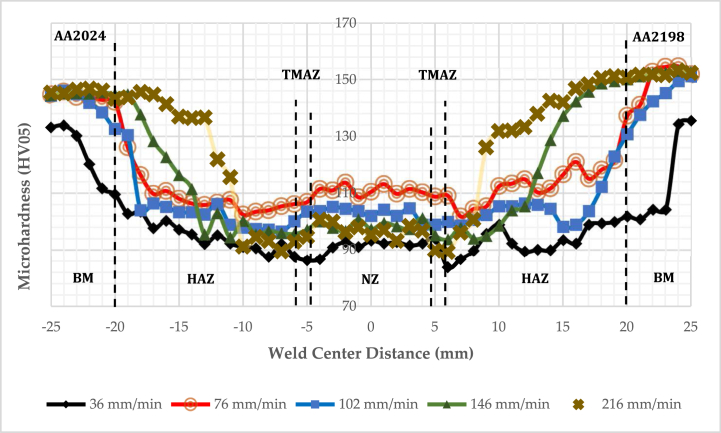
Microhardness of single-sided friction stir welding SS-FSW.

Vickers hardness HV profile of the SS-FSW and reversed DS-FSW fabricated joints by various running welding speeds presents the morphology and grain size changes. According to standard practice ASTM E 92, the HV05 was measured. Similarly, the HV05 profile distribution for the fabricated hybrid joints specified as a “W" shape gradually reduced in TMAZ and HAZ regions. The HAZ of the two welding processes at different running speeds specified the lowest HV05 measurement. Furthermore, the parent metal's Vickers hardness HV05 amount was higher than the three-weldment zones measured amount. Thus, it established that the three distinct welding zones had become softer, NZ, TMAZ, and HAZ. The dissolved individual particles that strengthen Al alloys during the FSW process are the critical reasons that lower the HV values of the NZ. However, the effect of the pin rotation in the stir zone caused the dynamic recrystallization; it precipitates and forms refined equiaxed grains in the NZ. Vickers hardness measured values in the two zones HAZ and TMAZ is smaller than the NZ, as presented in Fig. 6, Fig. 7. Especially in the welding running speeds of 102 mm/min for reversed DS-FSW, the typical HV value is higher due to refined equiaxed grain forms in the NZ. However, due to the formation of the coarse grains of the fabricated joint by 36 mm/min welding running speed, the lowest HV value was observed [24]. The HV result is a vital indication of the grain size and structure change.

### 3.2 Exfoliation corrosion

#### 3.2.1 Macrostructure examination

**Fig. 8:**
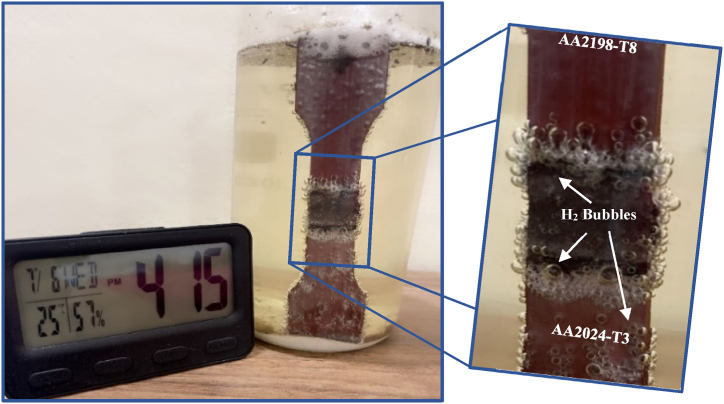
Exfoliation corrosion test for a tensile sample of DS-FSW at 102 mm/min welding speed.

**Fig. 9:**
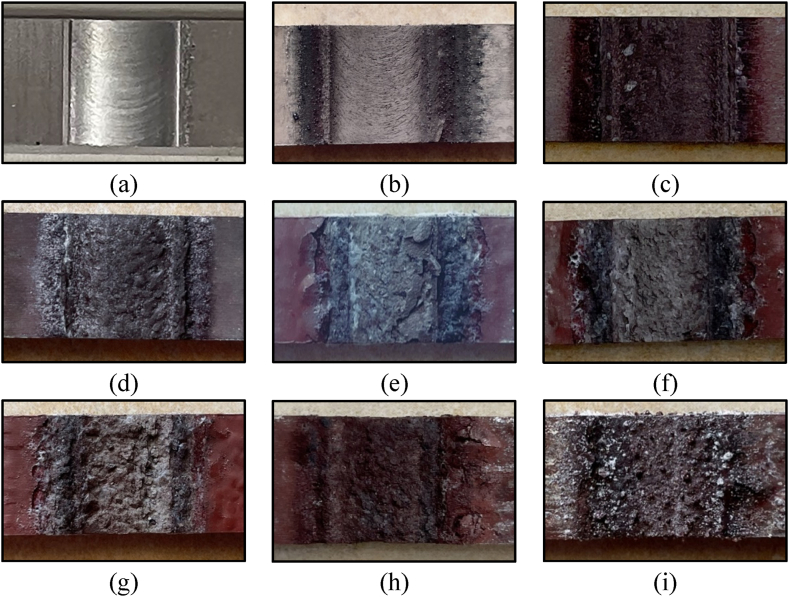
Macrostructure of the utmost efficient joint at various EXCO exposure times (a) As welded, (b) 15 h, (c) 30 h, (d) 45 h, (e) 60 h, (f) 75 h, (g) 90 h, (h) 105 h, and (i) 10 h.

The comparison corrosion test for Al–Li–Cu alloys AA2198-T8 against Al–Cu alloys AA2024-T3 was reported by Georgoulis et al. and they concluded that Al–Li–Cu alloys have better corrosion resistance than conventional Al–Cu alloys [17]. Tensile specimens were prepared and immersed in EXCO solution for eight different exposure aging times (15, 30, 45, 60, 75, 90, 105, and 120 h). Fig. 8 shows that the intense evolution of hydrogen gas (H_2_) in the TMAZ and the base material of aluminum alloy AA2024-T3 region leads to corrosion. With this H_2_ evolution in TMAZ at its free corrosion potential, it is evident that the TMAZ region is highly susceptible to EXCO. Thus, due to the variation of the heat generated during friction stir welding processes, the morphology of the nugget zone NZ and the interface zone of NZ/TMAZ. The interpretation of the EXCO attack in the degree of severity results was classified in compliance with ASTM G34. Fig. 9 shows the macrostructure of the EXCO test at different exposure times for the most efficient hybrid joint obtained by reversed DS-FSW.

A visual rating of the EXCO specimens can be provided by examining Fig. 9b and c. The exfoliation rating is EA (superficial), with few pits observed. In Fig. 9 (d), where the joint surface has distinct layering, the exfoliation rating is EB (moderate). Fig. 9 (e and f) show an exfoliation rating of EC (severe) owing to significant penetration into the joint surface. For Fig. 9 (g – i), the exfoliation rating is ED (very severe), similar to EC, though the penetration is more profound, with a metal loss of nearly 30% from the joint surface. The surface damage is limited for the short exposure intervals as no pits were observed after 45 h of exposure. However, pitting density and size tend to increase as exposure time increases; corrosion damage in the form of pits was found after 60 h of exposure, while additional pits with larger diameters and pit coalescence were observed after 90 h of exposure. With time, there is a deterioration in the surface of the tensile specimen of the hybrid joint welded using the DS-FSW process.

#### 3.2.2 Mechanical characteristics

**Fig. 10:**
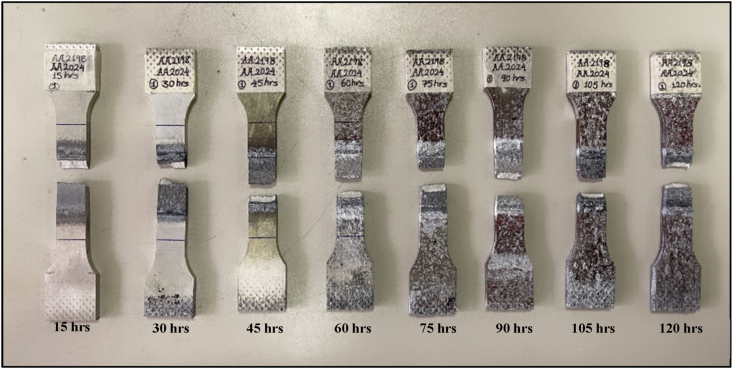
Tensile samples for different exposure times for exfoliation corrosion test.

**Fig. 11:**
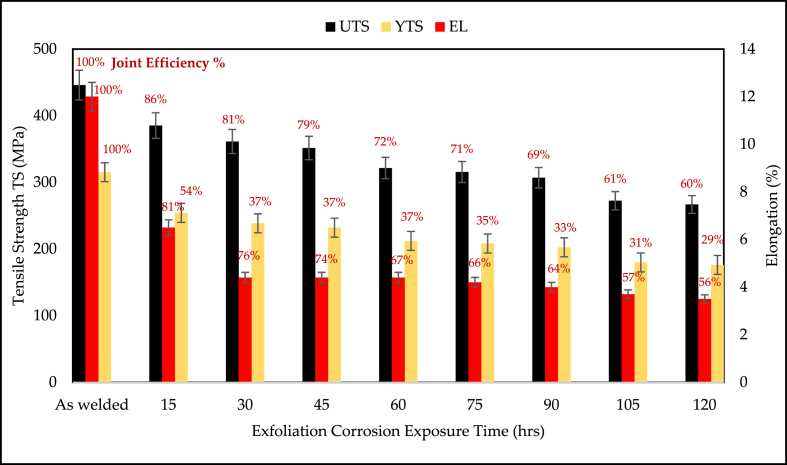
Mechanical characteristics of tensile samples after the different durations of exposure to exfoliation corrosion.

Fig. 10 shows the fractured tensile specimens for various aging times of EXCO. It is evident from Fig. 8 that the severe attack of H_2_ bubbles is localized and more concentrated in TMAZ and HAZ. This H_2_ bubble causes both hydrogen embrittlement and pit formation at the joint, which is localized at TMAZ. Fig. 11 shows the effect of EXCO on the mechanical properties of the reversed DS-FSW efficient hybrid joint for different exposure times. It is evident from Fig. 11 that when the exposure time of EXCO increases, it leads to a degradation in the tensile properties. However, comparing the utmost efficient joint in SS-FSW at 76 mm/min welding speed with the reversed DS-FSW at 102 mm/min welding speed remains irresistible until 60 h exposure time to EXCO even the efficiency drops of UTS 28% of the joint. The EXCO attack test shows that the durability of the reversed DS-FSW is another advantage of the technique over conventional SS-FSW. Thus, material recycling and joint treatment required for maintenance and replacement will be carried out in the longer term to reach a 40% joint efficiency drop of UTS for 120 h of EXCO exposure time.

#### 3.2.3 Metallographic examination

**Fig. 12:**
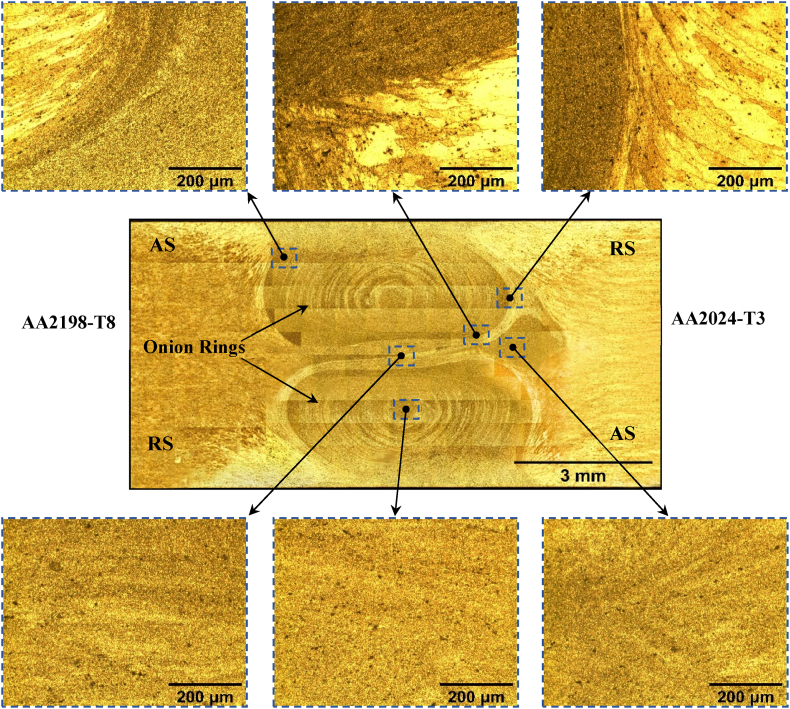
Microstructure of the as-welded reversed DS-FSW joint at 102 mm/min welding speed.

Fig. 12 shows the microstructure of the as-welded utmost hybrid joint for an unaltered tool rotational speed of 580 rpm at 102 mm/min welding speeds. Subsequently, reveal the microstructure of the hybrid joint by double etching solutions. The pictures were captured under a magnification of 345 for microstructure examination. The configured core microstructure is composed of 374 captures. As a result of the recrystallization behavior in the reversed DS-FSW, the grain size distribution in the SZ exhibited refined equiaxed grains. This region is closely correlated with the path of the pin during welding. The material's grain size in this zone is extremely small compared to the base metal. In this region of the weld, the microstructure comprises elements of both metals. This zone comprises multiple concentric rings on both sides and is called an “onion ring structure.” The onion ring shape is due to the different helically oriented grain size distributions. The TMAZ is located on both sides of the stir zone. Thus, the semi-cylindrical layers of the material are extruded with each tool revolution. These layers cool more quickly than the layer that follows them. The method of friction welding causes plastic deformation of the base metal. The strain and temperature are lower than in the SZ. Hence the influence of welding on the microstructure is less pronounced. The HAZ is formed in all welding processes due to the heat cycle of the weld. HAZ survives the heat cycle with no plastic deformation; grain growth occurs, and the hardness drop is noted from the hardness profile. Temperatures in the HAZ are lower than the TMAZ but still substantially affect the microstructure and mechanical behavior of the zone. Ralston et al. concluded that the larger grain size structure decreases the tendency of high-purity aluminum to corrosion in NaCl [25]. Therefore, progressive exfoliation corrosion can be seen in the TMAZ, and the exfoliation corrosion effect increases with exposure time.

#### 3.2.4 X-Ray Diffraction examination

**Fig. 13:**
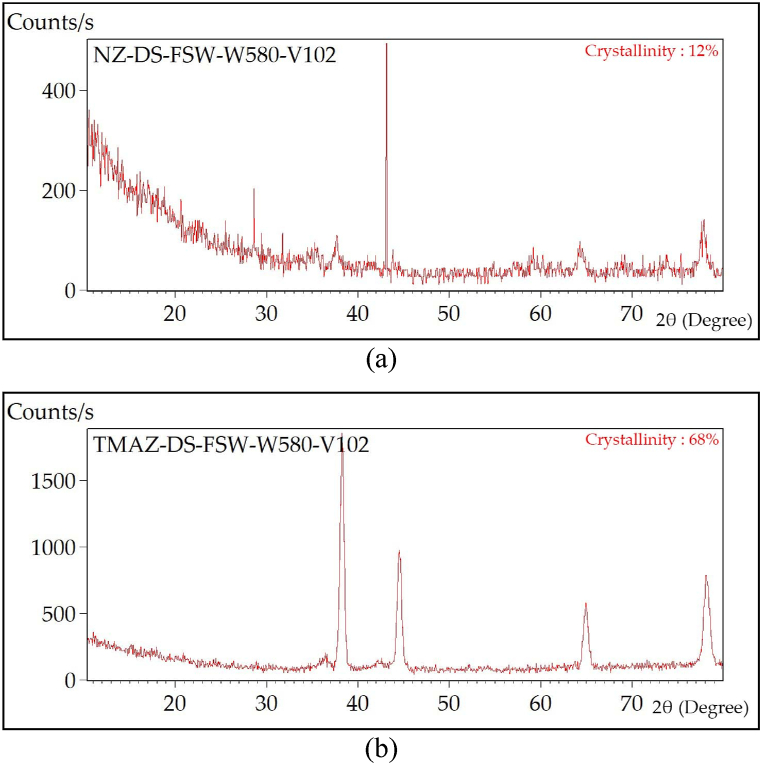
X-Ray Diffraction examination of as-welded reversed DS-FSW joint at 102 mm/min welding speed for (a) SZ and (b) TMAZ.

Fig. 13 shows the XRD results of the reversed DS-FSW benchmark hybrid joint at 102 mm/min welding speed. The XRD test was concentrated in the two central regions of the joint, SZ and TMAZ, as illustrated in Fig. 13 (a,b). The degree of crystallinity of the stir zone was 12%. The SZ is an 88% amorphous region with a smaller grain size in a cubic crystal structure. However, the degree of crystallinity in TMAZ was found to be 68%, and this zone of the joint is 32% amorphous, and grain sizes are significantly larger than the SZ. The degree of crystallinity and larger grain size structure affect the region's susceptibility to EXCO attack. XRD results demonstrate that the region most susceptible to EXCO attack was TMAZ.

#### 3.2.5 Fracture morphology examination

**Fig. 14:**
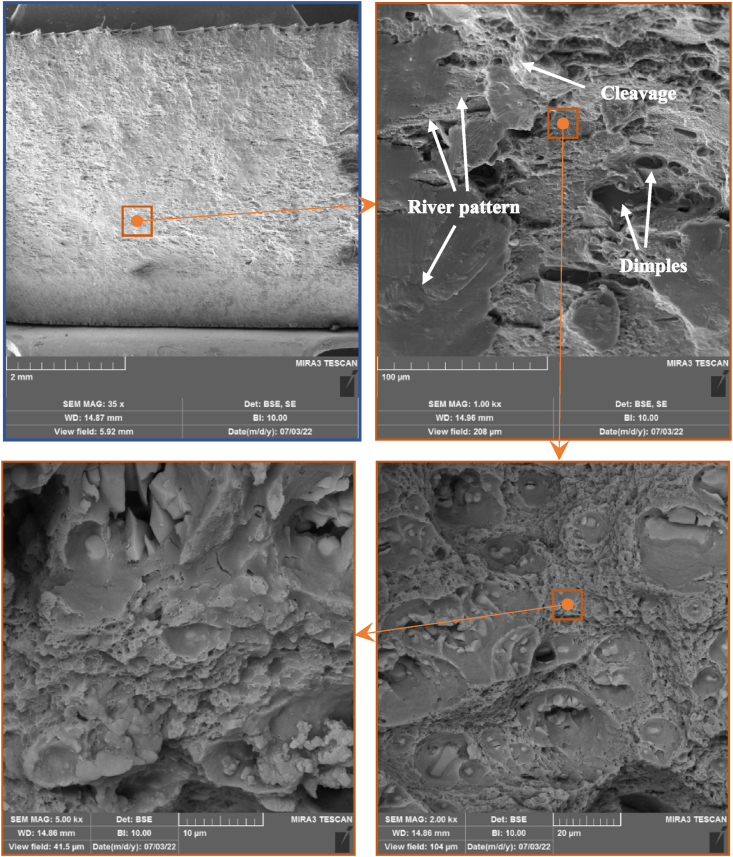
SEM examination of the fractured surface in the TMAZ region of as-welded reversed DS-FSW at 102 mm/min welding speed.

**Fig. 15:**
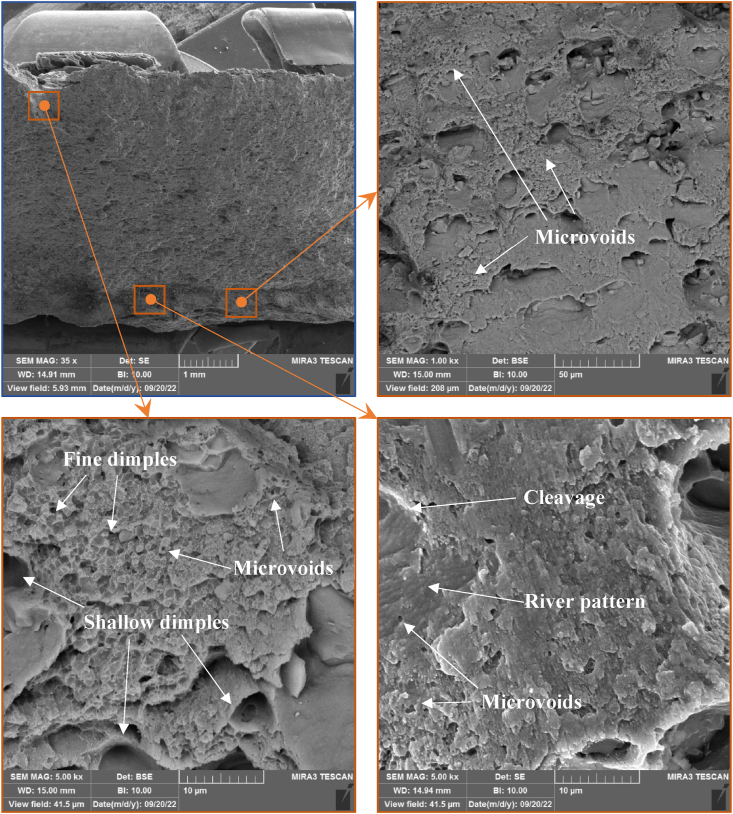
SEM examination of the corroded fractured surface in the TMAZ region for 120 h EXCO exposure time of reversed DS-FSW at 102 mm/min welding speed.

Fig. 14 illustrates the morphology SEM of the fractured surfaces of the as-welded tension joints resulting from specific welding speeds, giving the maximum hybrid joint efficiency and tensile properties. The fractured surface of the efficient hybrid joint reversed DS-FSW acquired at 102 mm/min has some dimples and cleavage platforms with some river patterns. It is a typical mixed fracture mode in the TMAZ region, indicating a typical mixed ductile and brittle fracture. Simultaneously, the degree of crystallinity of the TMAZ region indicates the fracture mode type. Moreover, the mixed crystal structure and larger grain sizes from the SZ appeared. Thus, the changes in microstructure and grain growth increase the probability of susceptibility to EXCO. Fig. 15 illustrates the SEM of the fractured surface of the EXCO test for 120 h exposure times. The microvoids result from the different grain sizes in the TMAZ due to dynamic recrystallization of the grain size transformation. Furthermore, the effect of these microvoids near the fine and shallow dimples led to weak fracture points in the tensile region. On the other hand, the appearance of these microvoids on the river pattern form, as represented by a brittle region, drops the strength of the joint.

## 4 Conclusions

The characteristic of both reversed DS-FSW and conventional SS-FSW has been examined in the case of tensile, macro-section examination, and microhardness outcomes. The utmost hybrid joint was the reversed DS-FSW at 102 mm/min welding speed. The EXCO examination has been elaborated upon for the most efficient hybrid joint of AA2024-T3 and AA2198-T8. The following conclusions were reached:

1. The joints produced at 102 mm/min exhibit superior tensile properties, such as an ultimate tensile strength of 446 MPa, yield strength of 315.2 MPa, and 12% elongation. Although, the revealed macro-section to sound weld and microhardness test showed that reversed DS-FSW provides superior mechanical properties compared to conventional SS-FSW.
2. Degradation in tensile properties was noticed in the most efficient hybrid joint region of AA2024-T3 to AA2198-T8 welded using reversed DS-FSW. However, the most efficient hybrid joint for conventional SS-FSW was not comparable to the joint efficiency of reverse DS-FSW in spite of the efficiency reduction of UTS being 28% after 60 h exposure to EXCO, considering the addition of the efficiency drop of the as-welded hybrid joint to the base metal AA2024-T3. Material recycling and joint treatment required for maintenance and replacement will be done in the longer term to reach a 40% joint efficiency drop of UTS at 120 h of EXCO exposure time.
3. The most susceptible region to EXCO was found in the TMAZ. The severe attack of H_2_ bubbles was localized and more concentrated in the TMAZ. These H_2_ bubbles cause both hydrogen embrittlement and pit formation at the joint, which is localized in the TMAZ. HAZ temperatures are lower than the TMAZ but significantly influence the zone's microstructure and mechanical behavior. Grain growth and progressive exfoliation corrosion are observable in the TMAZ, and the extent of exfoliation corrosion increases over time.
4. XRD results demonstrated that the region vastly susceptible to EXCO attack was TMAZ. The degree of crystallinity and larger grain size structure affect the susceptibility and the effect of attack by EXCO in that region.
5. In the SEM examination of the most efficient hybrid joint for the fractured TMAZ location surface and the 120 h EXCO specimen, mixed mode tensile fracture was found, indicating a typical mixed ductile and brittle fracture. In comparison, a ductile fractured mode surface indicates a large grain structure and a rise in the degree of crystallinity. Thus, the clustered hydrogen bubbles indicate the high susceptibility of the TMAZ region to the EXCO.

## Author contribution statement

Ahmed Samir A. Alemdar: Performed the experiments; Analyzed and interpreted the data; Wrote the paper.

Shawnim R. Jalal : Conceived and designed the experiments.

Mohammedtaher M. Mulapeer: Contributed reagents, materials, analysis tools or data.

## Data availability statement

Data included in article/supp. material/referenced in article.

## Declaration of competing interest

The authors declare that they have no known competing financial interests or personal relationships that could have appeared to influence the work reported in this paper.
